# Application of the comet assay in erythrocytes of *Oreochromis niloticus* (Pisces): A methodological comparison

**DOI:** 10.1590/S1415-47572009005000020

**Published:** 2009-01-23

**Authors:** Cintya A. Christofoletti, José Augusto O. David, Carmem S. Fontanetti

**Affiliations:** 1 Departamento de Biologia, Instituto de Biociências, Universidade Estadual Paulista Júlio de Mesquita Filho, Rio Claro, SPBrazil; 2 Departamento de Genética, Instituto de Biociências, Universidade Federal do Pará, Belém, PABrazil

**Keywords:** comet assay, ethidium bromide, silver staining, tilapia

## Abstract

The present study applied the comet assay to erythrocytes of *Oreochromis niloticus* with the aim of improving protocols to detect DNA damage in these cells, by using two distinct pHs (pH = 12.1 and pH > 13) and evaluating whether there is a correspondence between silver and ethidium bromide staining. Comets were visually examined and, the frequency of cells with and without damage was obtained, as well as the distribution of classes and scores. By using the Kruskal-Wallis test, our results revealed that pH 12.1 is more effective, although both pHs can be used. Our findings also suggest that silver staining can substitute ethidium bromide, an expensive and highly toxic stain that requires specific equipment for examination.

The development of new methodologies and the application of more sensitive assays to detect genotoxicity in different samples have been the subject of several scientific studies on environmental monitoring (Al-Sabti and Metcalfe, 1995; Lemos *et al.*, 2005). The Comet Assay, or single cell gel electrophoresis, is a sensitive genotoxicological method for assessing DNA damage in single cells, allowing for the quantification of DNA breaks and alkaline labile sites. Compared with other genotoxicity tests, the advantages of the comet assay are the detection of slight DNA damage, the low number of cells required, low cost, precision, ease of application, reproducibility, and short period of time to conduct the experiment (Belpaeme *et al.*, 1998; Tice *et al.*, 2000; Bücker *et al.*, 2006).

This technique is the result of studies undertaken by Östling and Johanson, who developed the methodology of DNA electrophoresis in micro-gel, and those by Singh *et al.* (1988), who improved the technique. Currently, several international research groups have published recommendations describing protocols and criteria for the comet assay, aiming at establishing high standards to obtain valid, reproducible, and accurate data (Klaude *et al.*, 1996; Brendler-Schwaab *et al.*, 2005; Di-Paolo, 2006).

The comet assay has been successfully applied in erythrocytes of several fish species, thereby showing the sensitivity of the blood cells of these animals to genotoxic effects (Padrangi *et al.*, 1995; Belpaeme *et al.*, 1998; Gontijo *et al.*, 2003). Thus, the aim of the present study was to apply the comet assay to erythrocytes of specimens of the fish *Oreochromis niloticus*, in order to improve protocols for analyzing DNA damage in these cells, by using two distinct pHs, and evaluate whether there is a correspondence between silver and ethidium bromide staining.

For the bioassay, two 20 L aquaria were filled with water from an artesian well, this then being aerated for 48 h and maintained at a room temperature ranging from 20 to 23 °C, prior to the experiment. Five specimens of *Oreochromis niloticus* (Perciformes, Cichilidae), commonly known as Nile tilapia, were placed in the aquaria (table 1). Fishes were obtained from a fish tank of the Institute of Biosciences of the São Paulo State University, Rio Claro campus. The fishes were not fed during the experiment and were exposed to a 14:10 h light/dark cycle, under constant aeration. These were then divided into two groups, treated and non-treated. Non-treated fishes were injected intraperitonially with saline solution (30 mL of physiological solution/50 g of fish) 72 h prior to the end of the experiment. The treated group received an intraperitonial injection of cyclosphosphamide (20 mg/mL) (30 mL of cyclosphosphamide /50 g of fish), also 72 h prior to the end of the experiment.

The comet assay was undertaken according to Singh *et al.* (1988) and Villela *et al.* (2006), with certain modifications, as follows. Slides were previously covered with 1.5% normal melting point agarose. Cardiac punctures were performed and 0.3 mL of blood was drawn from the specimens. The assay was done in triplicate and later evaluated regarding the effect of different pHs and staining. After cardiac punctures, 5 μL of the blood sample were diluted in 1000 μL of PBS. 10 μL of cell suspension with 120 μL of 0.5% low melting point agarose at 37 °C, were layered on previously prepared slides, cover-slipped and placed in the refrigerator for 5 min, for solidification of the gel. The cover-slips were then removed and the slides immersed in a freshly-prepared lysis solution (1 mL of Triton-X 100, 20 mL of DMSO and 79 mL of lysis stock solution: 2.5 M NaCl, 100 mM EDTA and 10 mM Tris, pH 10.0-10.5) for a minimum of at least one hour and a maximum of two, at 4 °C. After lysis, slides were transferred to a horizontal electrophoresis tank. For the first two runs, alkaline solution was added (300 mM NaOH and 1 mM EDTA, pH = 12.1) for 20 min. Upon completion of the relaxing time, slides were subjected to electrophoresis for 15 min at 21 V and 270 mA (0.8 V.cm^-1^). The same procedures were followed for the last run, although an alkaline buffer (NaOH 300 mM and 1 mM EDTA, pH > 13) was used in this case. All procedures were carried out under indirect light. After electrophoresis, slides were carefully removed from the tank. Neutralization was performed through three baths of 5 min with a neutral buffer (0.4 M Tris-HCl, pH 7.5) to remove salts and detergents. Slides were then allowed to dry at room temperature.

For ethidium bromide staining (Figure 1A-C), slides were fixed with 100% ethanol for 10 min and allowed to dry at room temperature. These were then stained with an aqueous solution of ethidium bromide (0.02 mg/mL) before examination under a fluorescence microscope (Leica DMLB), filter B-3^4^ (excitation: i = 420 nM; barrier: I = 520 nM), magnification 1000x.

For silver staining (Figure 1D-F), slides were immersed in a fixative solution (15% trichloroacetic acid, 5% zinc sulfate, 5% glycerol), for 10 min in a vertical tank, rinsed three times with distilled water and allowed to dry overnight at room temperature. They were then rehydrated for 5 min with distilled water and stained with 36 mL of solution A (5% sodium carbonate) and 54 mL of solution B (0.1% ammonium nitrate, 0.1% silver nitrate, 0.1% silicotungstic acid, 0.15% formaldehyde, freshly prepared and in the dark) under constant shaking at 37 °C for 10 min, or until they became either gray or brown. After staining, the slides were washed twice with distilled water and immersed in a stop solution (1% acetic acid) for 5 min, to be then washed again three times with distilled water and after allowed to dry at room temperature. They were later examined under a light microscope at 400x magnification.

One hundred comets per slide were examined using visual classification based on the migration of DNA fragments of the nucleus. Comets were classified into four classes: class 0 (no damage), class 1 (little damage), class 2 (medium damage), and class 3 (extensive damage). Data were presented as the frequency of damaged cells, class distribution, and damage scores, calculated as the sum of cells in each class and the total number of cells in each class multiplied by the number of classes (0-3). Scores ranged from zero (all cells with no damage - 0x100) to 300 (all cells with maximum damage 3x100), according to Rigonato *et al.* (2005). Statistical analysis was performed using the Kruskal-Wallis test, with the level of significance set at p < 0.05.

The results obtained here for the comet assay in *Oreochromis niloticus* erythrocytes showed similar images (Figure 1) and score (Table 2) for both staining procedures when pH 12.1 was used in the assay.

In the untreated group, a cell statistical difference was observed between pH 12.1 and pH > 13 (Table 2). When pH > 13 was used in this group, the number of class 0 comets decreased while class 1 comets revealed a significant increase (Table 2). This can probably be due to, the expression of alkali-labile sites that occur only with pH > 13.

Similar levels of damage were registered in treated group cells at pH 12.1 and pH > 13 (Table 2). The effect of treatment could be measured with both staining and pH, although the difference between treated and untreated groups was greater at pH 12.1, probably because of the expression of different DNA damage in pH > 13.

According to Padrangi *et al.* (1995) and Lemos *et al.* (2005), different tissues of aquatic organisms can be used for the comet assay. However, in most cases, erythrocytes have been used as target-cells, as they require small volumes that can be obtained through a non-damaging technique and cell dissociation is not needed (Belpaeme *et al.*, 1998). Gontijo *et al.* (2003) also applied the comet assay to erythrocytes of *Oreochromis niloticus* to assess possible modulation of induced DNA damage by benzocaine, in erythrocytes exposed to two known mutagens, (methyl methanesulfonate and hydrogen peroxide). Their findings suggested that benzocaine does not interfere with comet assay results and can be recommended for the welfare of the animal, thus preventing stress.

Souza and Fontanetti (2007) used erythrocytes of *O. niloticus* for the comet assay to detect possible genotoxicants in the waters of the Paraíba do Sul River in an area affected by an oil refinery. The authors concluded that substances with genotoxic potential were present in the waters of the river, as a frequency increase in class 2 and 3 nucleoids was observed in all the months of collection.

According to Belpaeme *et al.* (1998), the comet assay can also be used to complement studies of chromosome aberrations, micronuclei, and sister chromatid exchanges. Our findings revealed that blood cells of *O. niloticus* used in the comet assay can be used as genotoxic indicators, thus constituting an efficient, fast, and sensitive method to detect DNA damages in these cells.

Since being described, single cell gel electrophoresis has been used by researchers in a wide variety of organisms and tissues, thereby improving its usefulness. Thus, to better understand the effects of different pHs on the erythrocytes of tilapias, certain changes were made, based on studies by Tice *et al.* (2000), Gontijo *et al.* (2003) and David (2007), using pH 12.1, unwinding time equivalent to 20 min and electrophoresis time of 15 min, 21 V and 270 mA. pH variation during the lysis and electrophoresis steps affect the type of DNA breaks that can be detected, as DNA denatures and unwinds at pHs of around 12, due to breaks in hydrogen bridges between DNA strands (Kohn, 1991).

At pH 12.3, breaks in the double and single strands are detected, as well as alkali-labile sites or abasic sites (Lee and Steinert, 2003). According to Tice *et al.* (2000), pH > 13 is widely used to maximize the expression of alkali-labile sites and single-strand breaks and thus it is the most recommended, especially for vertebrate cells. However, it is worth pointing out that there is a wide variety of DNA lesions. Among these, single-strand breaks, do not give rise to drastic effects, as they are quickly repaired, whereas double stranded DNA breaks are much more severe and have much more complex repair pathways (Gontijo *et al.*, 2003; Moller, 2006; David, 2007).

Di-Paolo (2006) evaluated the different exposure times of erythrocytes in snooks during the various steps in lysis and electrophoresis, and observed that there was a gradual increase in the intensity of DNA migration due to the effects from an increase in voltage of electrophoresis on the morphology of comets. The author also concluded that the best results were obtained at pH 12.6 and pH > 13, under the following conditions: 10 min for unwinding and, 20 min for electrophoresis at 0.8 V/cm and 300 mA, thereby indicating the suitability of the assay for measuring damage in the blood cells of snooks.

Our results revealed that pH 12.1 as well as pH > 13 are effective for the comet assay in tilapia erythrocytes. Although many authors, such as Tice *et al.* (2000) and Gontijo *et al.* (2003) suggested a pH > 13 for both vertebrate cells and *O. niloticus*, pH 12.1 was the most efficient in this study, as there was a greater difference between treated and untreated cells on using pH 12.1 when compared to pH > 13 (Table 2).

Ethidium bromide is traditionally used for staining materials examined by fluorescence. However, this dye has certain negative aspects, such as its toxic and mutagenic effects, the need for specific equipment and the short duration of staining. Silver staining, on the other hand, has been described as a good alternative for the comet assay, demonstrating high sensitivity when compared to ethidium bromide (Reinhardt-Poulin *et al.*, 2000; Di-Paolo, 2006).

Di-Paolo (2006) applied silver staining in a comet assay on the blood cells of snooks and found high sensitivity when visually examining comets, as this is a fast and simple method that can be observed under the light microscope. Villela *et al.* (2006) obtained similar results in a study undertaken with the bivalve *Limnoperna fortune* when using different concentrations of copper sulfate and pentachlorophenol.

Garcia *et al.* (2007) described a protocol for silver staining when using the comet assay in human lymphocytes exposed to gamma radiation, and obtained a significant percentage of DNA in the tail of silver-stained comets, very similar to those obtained by fluorescence staining. Other parameters of comets, such as length and momentum of the tail, were also quantified using conventional microcopy and Internet software.

The results obtained in our study showed that both stains were efficient. Silver staining, however is recommended, as it is less toxic than ethidium bromide, does not require specific equipment for analysis, and provides long lasting staining.

Thus, this study suggests that erythrocytes of *Oreochromis niloticus* are good genotoxicity indicators through the comet assay, an efficient, fast, and sensitive method to detect DNA damage. Our data also revealed that pH 12.1 is the best, although both pHs, 12.1 and pH > 13 can be used with adaptations under electrophoresis conditions. Moreover, silver staining is recommended in preference to ethidium bromide.

**Figure 1:**
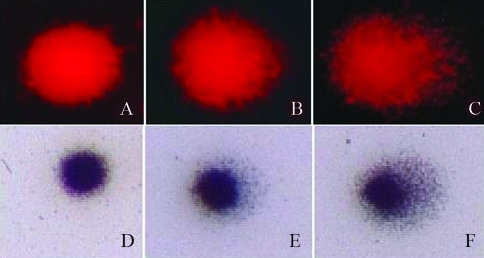
Comet assay applied in erythrocytes of *O. niloticus* using pH 12.1 stained with ethidium bromide (A. Class 0, B. Class 1, C. Class 2) and silver (D. Class 0, E. Class 1, F. Class 2).

## Figures and Tables

**Table 1:** Mean and standard deviation of the weight and size of fishes used in the comet assay for both treatments.

Treatments	Weight (g)	Size (cm)
Non-treated	37.1 ± 10.60	13.24 ± 1.66
Treated	41.34 ± 8.01	13.98 ± 0.82

**Table 2:** Average number of cells with comet, distribution of classes and damage scores (mean and standard deviation) for the two treatments using two pHs and forms of staining.

Treatments	pHs	Staining	NA	Comets	Classes				Score
					0	1	2	3	
Non-treated	12.1	silver	500	10.6 ± 1.4^*a, b^	89.2 ± 1.4^*c^	9.4 ± 0.8^*d^	1.2 ± 0.4	0	11.8 ± 1.48^*a, b^
		ethidium bromide	500	18.8 ± 1.92^*a^	81.4 ± 1.6	18.6 ± 1.6	0.2 ± 0.4	0	19 ± 2.2^*a^
	> 13	silver	500	46.25 ± 11.20^*b^	54.2 ± 10.2^*c^	39 ± 10.09^*d^	4.75 ± 1.25	2.5 ± 1.2	56 ± 13.31^*b^

Treated	12.1	silver	500	81.2 ± 6.45	18.8 ± 6.4	79 ± 5.8	2.2 ± 1.7	0	83.4 ± 7.4
		ethidium bromide	500	87 ± 3.53	15 ± 1.5	86.2 ± 3.2	0.8 ± 0.8	0	87.8 ± 3.9
	> 13	silver	500	87.8 ± 6.97	12.2 ± 6.9	67 ± 13.5	14.6 ± 9.9	6.2 ± 2.2	108.8 ± 9.20

*Identical letters: significant difference using the Kruskal-Wallis test (p < 0,05). NA: total nucleoids analyzed.

## References

[Al-SabtiandMetcalfe1995] Al-Sabti K., Metcalfe C.D. (1995). Fish micronuclei for assessing genotoxicity in water. Mutat Res.

[BelpaemeandCooremanKandKirsch-Volders1998] Belpaeme K, Cooreman Kand, Kirsch-Volders M (1998). Development and validation of the *in vivo* alkaline comet assay for detecting genomic damage in marine flatfish. Mutat Res Genet Toxicol Environ Mutagen.

[Brendler-Schwaabetal2005] Brendler-Schwaab S., Hartmann A., Pfuhler S., Speit G. (2005). The *in vivo* comet assay: Use and status in genotoxicity testing. Mutagenesis.

[Buckeretal2006] Bücker A., Carvalho W., Alves-Gomes J.A. (2006). Avaliação da mutagênese e genotoxicidade em *Eigenmannia virescens* (Teleostei, Gymnotiformes) expostos ao benzeno. Acta Amazônia.

[Garciaetal2007] Garcia O., Romero I., Gónzalez J.E., Mandina T. (2007). Measurements of DNA on silver stained comets using free Internet software. Mutat Res.

[Gontijoetal2003] Gontijo A.M.M.C., Barreto R.E., Speit G., Reyes V.A.V., Volpato G.L., Salvadori D.M.F. (2003). Anesthesia of fish with benzocaine does not interfere with comet assay results. Mutat Res.

[Klaudeetal1996] Klaude M., Eriksson S., Nygren J., Ahnström G. (1996). The comet assay: Mechanisms and technical considerations. Mutat Res.

[Kohn1991] Kohn K.W. (1991). Principles and practice of DNA filter elution. Pharmacol Ther.

[LeeandSteinert2003] Lee R.F., Steinert S. (2003). Use of the single cell gel electrophoresis/comet assay for detecting DNA damage in aquatic (marine and freshwater) animals. Mutat Res.

[Lemosetal2005] Lemos N.G., Dias A.L., Silva-Souza A.T., Mantovani M.S. (2005). Evaluation of environmental waters using the comet assay in *Tilapia rendalli*. Environ Toxicol Pharmacol.

[Moller2006] Moller P. (2006). The alkaline comet assay: Towards validation in biomonitoring of DNA damaging exposures. Pharmacol Toxicol.

[Padrangietal1995] Padrangi R., Petras M., Ralph S., Vrzoc M. (1995). Alkaline single cell gel (comet) assay and genotoxicity monitoring using bullheads and carp. Environ Mol Mutagen.

[Reinhardt-Poulinetal2000] Reinhardt-Poulin P., McLean J.R., Deslauriers Y., Gorman W., Cabat S., Rouabhia M. (2000). The use of silver-stained “comets” to visualize DNA damage and repair in normal and *Xeroderma pigmentosum* fibroblasts after exposure to simulated solar radiation. Photochem Photobiol.

[Rigonatoetal2005] Rigonato J., Mantovani M.S., Jordão B.Q. (2005). Comet assay comparison of different *Corbicula fluminea* (Mollusca) tissues for the detection of genotoxicity. Genet Mol Biol.

[Singhetal1988] Singh N.P., McCoy M.T., Tice R.R., Schneider E.L. (1988). A simple technique for quantification of low levels of DNA damage in individual cells. Exp Cell Res.

[Ticeetal2000] Tice R.R., Agurell E., Anderson D., Burlinson B., Hartmann A., Kobayashi H., Miyamae Y., Rojas E., Ryu J.C., Sasaki Y.F. (2000). Single cell gel/comet assay: Guidelines for *in vitro* and *in vivo* genetic toxicology testing. Environ Mol Mutagen.

[Villelaetal2006] Villela I.V., Oliveira I.M., Silva J., Henriques J.A.P. (2006). DNA damage and repair in haemolynph cells of golden mussel (*Limnoperna fortunei*) exposed to environmental contaminants. Mutat Res.

